# A comparison between ultrasound-guided AIIS injection and radiography in the diagnosis of subspine impingement in patients with FAI

**DOI:** 10.1186/s12891-022-06045-7

**Published:** 2022-12-12

**Authors:** Zi-Yi He, Zi-Ming Liu, Gui-Juan Bi, Xiao-Le Zhang, Jian-Quan Wang, Ling Jiang, Xiao-Dong Ju

**Affiliations:** 1 Department of Sports Medicine, Peking University Third Hospital, Institute of Sports Medicine of Peking University, Beijing Key Laboratory of Sports Injuries, 49 North Garden Rd, Haidian District, Beijing, 100191 People’s Republic of China; 2 Peking University Health Science Center, 38 Xueyuan Rd, Haidian District, Beijing, 100191 People’s Republic of China; 3 Department of Diagnostic Ultrasound, Peking University Third Hospital, 49 North Garden Rd, Haidian District, Beijing, 100191 People’s Republic of China

**Keywords:** Subspine impingement, Ultrasound-guided AIIS injection, AIIS morphology, Superior capsular oedema

## Abstract

**Background:**

Subspine impingement (SSI) does not have effective diagnostic criteria, especially in patients who also have femoroacetabular impingement (FAI). The classification of anterior inferior iliac spine (AIIS) morphology via three-dimensional CT is controversial.

**Purpose:**

To propose a method for ultrasound-guided AIIS injection as a way to diagnose SSI and evaluate the accuracy of radiography methods, including 3-D CT and MRI, as well as intraoperative findings.

**Methods:**

Patients diagnosed with FAI between September 2020 and December 2021 were evaluated in this prospective study. Those who met the criteria were included in the ultrasound-guided AIIS injection test. Whether the pain was relieved after injection was recorded in the radiology report. Patients who experienced significant relief of the anterior groin pain (more than 50%) after the AIIS injection were considered positive responders. Among these patients, radiography materials, including AIIS morphology as measured by 3-D CT as well as superior capsular oedema on MRI, were compared. The presence of congestion or bruising on the capsule side of the labrum corresponding to the AIIS during hip arthroscopy was recorded.

**Results:**

A total of 73 patients with FAI underwent the ultrasound-guided AIIS injection test. Prevalence rates of 13.70% (10/73), 58.90% (43/73), 23.29% (17/73) and 4.11% (3/73) were recorded for Type I, Type IIA, Type IIB and Type III AIISs, respectively. Thirty-six patients had positive responses to injection, and 37 patients had negative responses to injection. None of the patients with Type I, 23 (53.49%) patients with Type IIA, 11 (64.71%) patients with Type IIB and 2 (66.7%) patients with Type III AIISs had positive responses to the injection. A total of 57.14% of patients with Type II or Type III AIIS had positive responses to the injection. The proportions of patients with superior capsular oedema on MRI in the Type I, Type IIA, Type IIB, and Type III AIIS groups was 0, 30.23, 29.41 and 0%, respectively. Among non-Type I AIIS patients, those who reported positive responses to the injection had a higher incidence of superior capsular oedema (38.89% vs. 14.81%, *P =* 0.036), but they had no significant differences in the proportion of congestion or bruising of the labrum (47.22% vs. 37.04%, *P =* 0.419). The results showed that no pairs of methods—ultrasound-guided injection, MRI, and intraoperative findings—achieved good consistency (κ = 0.222, κ = 0.098 and κ = − 0.116).

**Conclusions:**

Radiographic methods including 3-D CT and MRI as well as the intraoperative findings of the labrum cannot be considered an accurate and reliable basis for the diagnosis and treatment of SSI in FAI patients. It is suggested that ultrasound-guided AIIS injections be combined with radiography to better diagnose SSI.

**Level of Evidence:**

IV, case series.

## Introduction

Hip impingement is a term that describes an abnormal mechanical conflict between the femur and acetabula and refers to both intra- and extra-articular forms of disease. Femoroacetabular impingement (FAI) is recognized as intra-articular hip impingement. More recently, various types of extra-articular hip impingement have also been described that can contribute to overall hip pain and dysfunction, one of which is subspine impingement (SSI) caused by hypertrophy of the anterior inferior iliac spine [1, 2]. FAI and SSI have similar clinical characteristics, manifesting as pain induced by the impingement produced by flexion, adduction, and internal rotation tests [3, 4], and are difficult to distinguish. Additionally, an SSI morphology can be observed concomitantly in patients with symptomatic FAI [5]. Increasing evidence has shown that arthroscopic treatment for AIIS deformities can achieve excellent outcomes [6, 7], especially for those who have both intra- and extra-articular forms of hip dysfunction, because it provides a chance for the surgeon to address all pathologies with one procedure without causing additional trauma [8].

However, failure to identify SSI sources of hip pain in FAI syndrome might lead to inferior outcomes following arthroscopy and potential risks for revision surgery [9]. Therefore, it is of great clinical significance to accurately identify patients with SSI. Hetsroni et al. previously proposed evaluating the AIIS morphology through three-dimensional CT and classifying the AIIS into three variants: Type I, Type II, and Type III [10]. Among them, the latter two types are usually related to SSI symptoms. Type II was further divided into Type IIA and Type IIB according to whether the AIIS protruded forward [11]. However, an increasing number of studies have suggested that symptomatic subspine impingement does not completely depend on AIIS morphology. Wong et al. reported that whether a patient had symptomatic hips was not consistent with the AIIS morphology of the patient [12]. Balazs et al. found that the positive predictive value of a Type II or Type III AIIS classification for impingement symptoms was only 10%. Both studies concluded that the current radiographic classification cannot be relied upon for the diagnosis of subspine impingement. In addition, signs of impingement of the distal femoral neck evaluated by MRI were also considered to be related to the diagnosis of SSI [13].

Objective and exclusive diagnostic criteria for SSI are lacking. There is no certain radiographical method for surgeons to know whether the AIIS is involved in the occurrence of hip pain and labrum injury in patients with FAI. Thus, in this study, we proposed using an ultrasound-guided AIIS injection for the diagnosis of SSI with extra-articular symptoms. We hypothesized that the method of ultrasound-guided AIIS injection can 1) be used to diagnose SSI and 2) prove that radiographic methods, including 3-D CT and MRI, cannot be considered accurate and reliable diagnosis and treatment bases for symptomatic SSI.

## Methods

### Patient Selection

We prospectively evaluated patients who were diagnosed with FAI between September 2020 and December 2021. The inclusion criteria were as follows: (1) have preoperative CT and MRI and a diagnosis of FAI; (2) complaints about hip pain in the groin in a typical C-region from anterior to posterior that could be reproduced by flexion, adduction, and internal rotation (FADIR) or flexion abduction external rotation (FABER) tests; and (3) pain for more than 6 months and ineffective conservative treatment for more than 3 months. The exclusion criteria were as follows: (1) neurologic symptoms or physical signs of the lower extremities, (2) diffuse idiopathic skeletal hyperostosis, (3) synovial chondromatosis of the hip, (4) osteoidosteoma of the hip, (5) previous hip surgery or (6) bilateral hips with FAI symptoms. In addition, severe deformity, severe anteversion or retroversion of the femoral head, severe instability, centre-edge angle less than 20, Tönnis grade more than 2, and joint space less than 2 mm were not indications for arthroscopy. The ethics committee of the institution approved this study.

### Ultrasound-Guided AIIS Injection

**Fig. 1:**
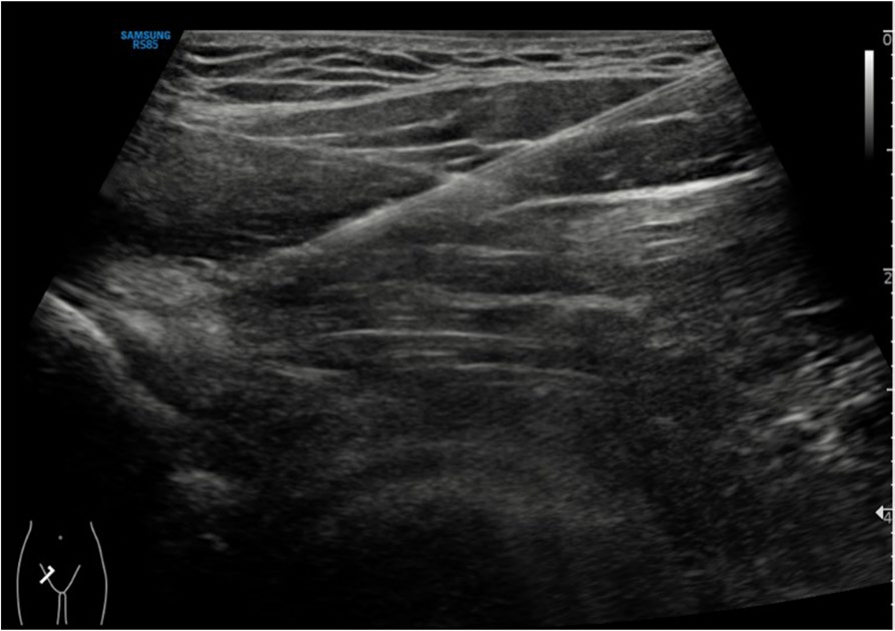
Imaging of Ultrasound Guided AIIS Injection

Ultrasound-guided injections were performed by a single radiologist who was blinded to both the results of 3D-scans and MRI scans and specializes in musculoskeletal disorders with more than 10 years of experience. Initial evaluation of the hip commenced with the patient in the supine position, and the probe was placed in the longitudinal plane in line with the AIIS at the level of the hip joint. The probe was swiped medially until the AIIS was identified. A 22-gauge 10-cm-long spinal needle was advanced into the AIIS and the origin of the direct head of the rectus femoris tendon and ilio-capsularis. As shown in Fig. 1, once the tip of the needle contacted the bone site, the extra-articular position of the needle was further confirmed by injecting 4 mL of 2% lidocaine. Then, a spot image was taken to document the location.

### Analysis of the Response to Injection

Before the injection, FADIR and FABER tests were examined by a sports medicine specialist, and the visual analogue scale (VAS) score and range of motion (ROM) of the hip were recorded. After the injection, patients were observed for at least 10 minutes, and pain relief was recorded in the radiology report. The response to the injection was analysed in terms of percentage relief of pain as assessed by the VAS score, with a “positive response” meaning more than 50% pain relief after injection, and a negative response meaning less than 50% pain relief or even pain aggravation. FADIR and FABER tests were performed, and the VAS score and ROM were recorded by the same doctor. We performed subspinal decompression in the process of FAI treatment by arthroscopy for patients with a positive response to the ultrasound-guided AIIS injection.

### Radiographic evaluation

**Fig. 2:**
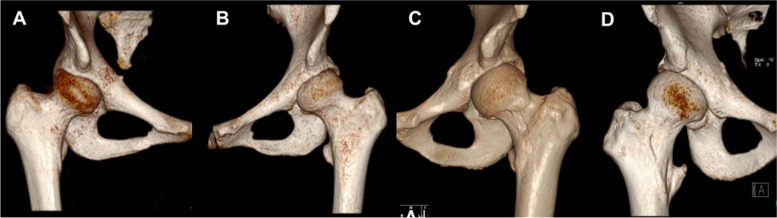
Type I (**A**), Type IIA (**B**), Type IIB (**C**) and Type III (**D**) AIISs Imaged by 3-D CT

**Fig. 3:**
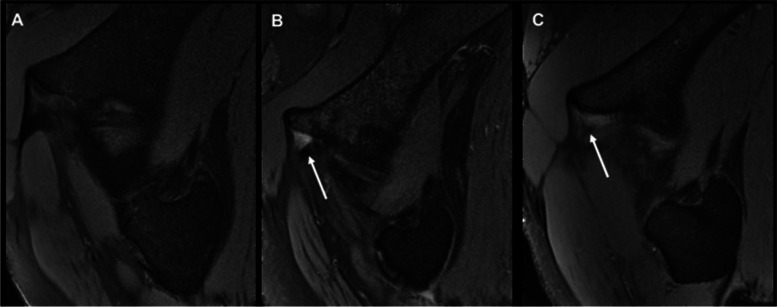
Normal Anterior Inferior Iliac Spine (**A**) and Superior Capsular Oedema in an AIIS on MRI (**B**-**C**)

Routine radiographic evaluation consisting of antero-posterior (AP) and modified Dunn views was performed for every patient. Protrusio acetabuli, the lateral centre-edge angle (LCEA) and crossover sign were assessed on AP view. The alpha angle was calculated on a modified Dunn view. AIIS morphology types were determined from 3D-CT scans (Fig. 2). We performed MRI first and then ultrasound. Other causes of hip pain were excluded by MRI, ultrasound, and physical examination. One radiologist specializing in musculoskeletal disorders with 10 years of experience in musculoskeletal radiology analysed all MRI scans and was blinded to the results of the ultrasound-guided AIIS injection (Fig. 3). All MRI examinations were performed using a previously published protocol on a 3.0-T MRI scanner (Magnetom Trio with TIM system; Siemens Healthcare, Malvern, PA) and a dedicated flexible surface coil around the affected hip joint.

### Surgical Procedures

**Fig. 4:**
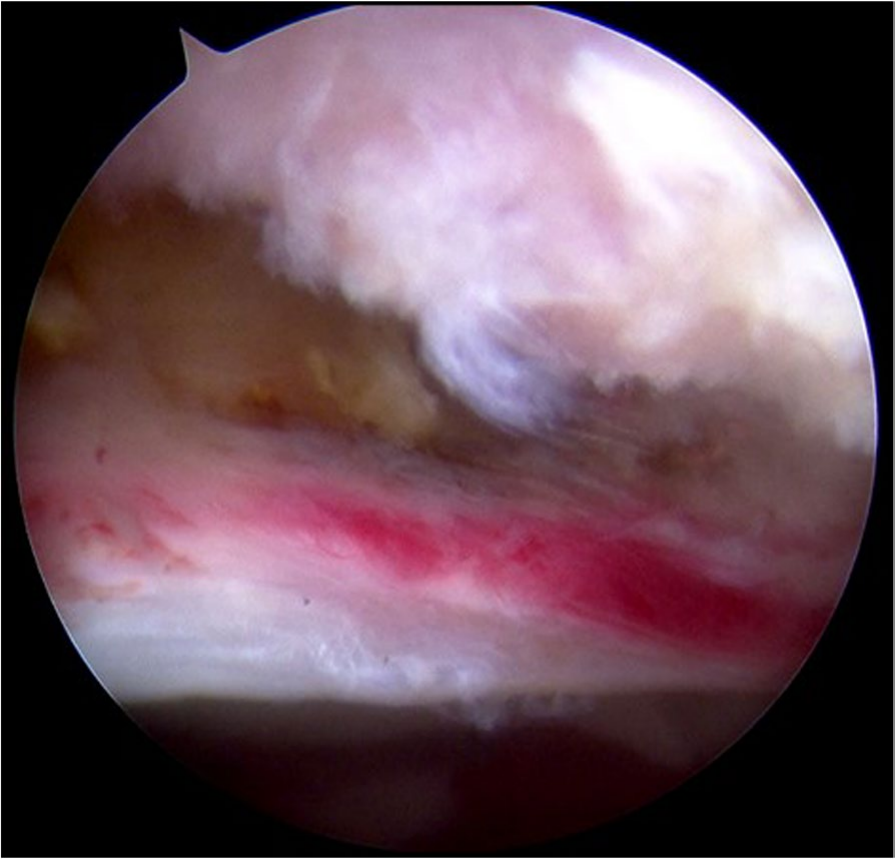
Intraoperative Findings of Congestion or Bruising on the Capsule Side of the Labrum in a Patient

One surgeon with more than 10 years of experience performed all hip joint arthroscopies. All patients underwent standard hip arthroscopy. After anaesthesia, the patient was placed in the supine position on standard hip traction (Smith & Nephew). Three standard portals were used: an anterolateral (AL) portal, a mid-anterior portal (MAP), and a proximal mid-anterior portal (PMAP). A detailed inspection of the central compartment was performed to assess the acetabular rim, acetabular labrum, articular cartilage, and ligamentum teres. As shown in Fig. 4, whether there was congestion or bruising on the capsule side of the labrum corresponding to AIIS labral repair or labral debridement was performed according to the nature of the injury was recorded. Femoral osteoplasty or acetabuloplasty was performed if cam or pincer lesions were identified. Cartilage damage was recorded according to the Outerbridge classification system. Capsular closure was routinely performed at the end of surgery. Subspinal decompression was performed on patients who had a positive response after the ultrasound-guided injection.

### Statistical Analysis

Categorical data were evaluated using the chi-square test. The agreement between different diagnosis methods was evaluated by calculating Cohen’s kappa. The level of agreement was defined as follows: poor (κ < 0.2), fair (0.2 < κ ≤0.4), moderate (0.4 < κ ≤0.6), substantial (0.6 < κ ≤0.8), and nearly perfect (κ > 0.8). All data were statistically analysed using SPSS 26.0 (IBM, Armonk, NY) software and are expressed as the mean ± standard deviation values. *P* values < 0.05 were considered statistically significant.

## Results

A total of 73 patients with FAI (40 male and 33 female), average age 35.27 ± 10.22 years (range 13–56), underwent the ultrasound-guided AIIS injection test. There were 30 left hips and 43 right hips. The mean alpha angle was 73.78° ± 12.00°, and the mean lateral centre-edge angle was 37.37° ± 8.12° (Table 1). The percentages of positive FADIR and FABER tests were 89.04 and 67.12%, respectively.

**Table 1:** Demographics of Patients Undergoing Ultrasound-Guided AIIS Injection ^*a*^

Patient demographics	
Patients eligible for study, n	73
Hips included	
Left	30 (41.10%)
Right	43 (58.90%)
Sex	
Female	33 (45.21%)
Male	40 (54.79%)
Age at surgery	35.27 ± 10.22
Preoperative α angle	73.78 ± 12.00
Preoperative LCEA	37.37 ± 8.12
FADIR test	
Positive	65 (89.04%)
Negative	8 (10.96%)
FABER test	
Positive	49 (67.12%)
Negative	24 (32.88%)

^*a*^ Values are presented as the mean ± SD or no. (%)

As shown in Fig. 2, all hips were classified according to the AIIS morphology: 10 hips were Type I (13.69%), 43 hips were Type IIA (58.90%), 17 hips were Type IIB (23.29%) and 3 hips were Type III (4.11%). A total of 36 (49.32%) patients had positive responses to the injection, and 37 (50.68%) patients had negative responses to the injection. None of the patients with a Type I AIIS, 23 (53.49%) patients with a Type IIA AIIS, 11 (64.71%) patients with a Type IIB AIIS and 2 (66.7%) patients with a Type III AIIS had positive responses to the injection. A total of 57.14% of patients with a Type II or Type III AIIS had positive responses to the injection (Table 2). The percentages of patients with superior capsular oedema on MRI among those with Type I, Type IIA, Type IIB, and Type III AIISs were 0, 30.23, 29.41 and 0%, respectively. Congestion or bruising of the labrum was observed in one (10%) Type I AIIS, 15 (34.88%) Type IIA AIIS, 9 (52.94%) Type IIB AIIS and 3 (100%) Type III AIIS patients (Table 2).

**Table 2:** Relationship Between AIIS Morphology and Response to Ultrasound-Guided AIIS Injection, Superior Capsular Oedema on MRI and Intraoperative Findings of Congestion or Bruising of the Labrum in the Patients^*a*^

AIIS morphology	I	II A	II B	III
	10 (13.70%)	43 (58.90%)	17 (23.29%)	3 (4.11%)
Positive response to injection	0 (0%)	23 (53.49%)	11 (64.71%)	2 (66.67%)
Superior capsular edema on MRI	0 (0%)	13 (30.23%)	5 (29.41%)	0 (0%)
Congestion or bruise of the labrum	1 (10%)	15 (34.88%)	9 (52.94%)	3 (100%)

^*a*^ Values are presented as no. (%)

Among non-Type I AIIS patients, those who reported positive responses to the injection had a higher incidence of superior capsular oedema (38.89% vs. 14.81%, *P =* 0.036), but there were no significant differences in the rates of intraoperative findings, including congestion or bruising on the capsule side of the labrum (47.22% vs. 37.04%, *P =* 0.419). Then, we analysed the concordance of any two of the three methods (ultrasound-guided injection, MRI, and intraoperative findings) in diagnosing patients with SSI. The results show that none of the pairs of methods good consistency in diagnosing SSI (κ = 0.222, κ = 0.098 and κ = − 0.116) (Table 3).

**Table 3:** Agreement Between the Diagnostic Ultrasound-guided Anterior Inferior Iliac Spine Injection Method, Superior Capsular Oedema on MRI and Intraoperative Findings of Congestion or Bruising of the Labrum in Patients with Type II and Type III AIISs

Response to injection	Positive(*n =* 36)	Negative(*n =* 27)	*P* value	κ Coefficient
Superior capsular edema on MRI (*n =* 18)	14	4	0.036	0.222
Without Superior capsular edema on MRI (*n =* 45)	22	23		
Response to injection	Positive(*n =* 36)	Negative(*n =* 27)	*P* value	κ Coefficient
Congestion or bruise of the labrum (*n =* 27)	17	10	0.419	0.098
Without congestion or bruise of the labrum (*n =* 36)	19	17		
Image findings	Superior capsular edema on MRI (*n =* 18)	Without Superior capsular edema on MRI (*n =* 45)	*P* value	κ Coefficient
Congestion or bruise of the labrum (*n =* 27)	6	21	0.334	−0.116
Without congestion or bruise of the labrum (*n =* 36)	12	24		

## Discussion

In this study, we proposed using an ultrasound-guided AIIS injection test to diagnose SSI. Although it is not possible to directly compare the advantages of this method with current imaging methods, we believe that ultrasound-guided injection of the AIIS can complement existing diagnostic methods and can better distinguish the source of pain in FAI patients. Our results also showed that using the ultrasound-guided AIIS injection may result in diagnostic results inconsistent with those of 3D-CT or MRI. In addition, we also found that intraoperative findings, MRI, or CT to diagnose SSI could not achieve consistent results.

Increasing evidence has proven that ultrasound-guided hip injection is convenient and produces little pain and can be used to locate the origin of pain [14–17]. Gao et al. reported that ultrasound-guided hip injections had high accuracy in diagnosing FAI and identifying intra-articular lesions in patients with atypical pain, even higher than MRI [18]. Amar et al. reported that ultrasound was reliable in assessing the morphology of the AIIS [19]. Previously, it was proposed that an anaesthetic injection into the subspine region can be diagnostic [20]. Recently, Xu et al. reported using extra-articular subspine corticosteroid injections to diagnose SSI. Patients who experienced significant relief underwent subspine decompression and achieved significant improvement [21]. Based on the existing evidence, we considered that using ultrasound-guided AIIS injections for the diagnosis of SSI with extra-articular symptoms would be feasible and effective.

After tentative application, we found that none of the patients with Type I AIISs reported a positive response. Hetsroni et al. considered that Type I AIISs were characterized by a lack of contribution to hip impingement [10]. Balazs et al. reported that only 20% of patients undergoing arthroscopic surgery for hip impingement had Type I AIIS [22]. Thus, the negative response reported by all of the patients with a Type I AIIS was in line with our expectations and proved that patients will not produce false-positive results due to the placebo effect in our method. In contrast, Aguilera-Bohorquez et al. reported that 52.2% of patients with both FAI and SSI were classified as having a Type I AIIS. However, in their study, the pain of the patients could not be established to determine whether it could be attributed to FAI or SSI [5]. In other words, they were uncertain if a Type I AIIS was involved in the appearance of symptoms. In our study, ultrasound-guided AIIS injections helped surgeons judge whether the source of pain and limitation of hip movement was primarily from the AIIS. The results of this study proved that a Type I AIIS did not contribute to the patient’s symptoms. Among a total of 73 patients, 36 reported a positive response after an ultrasound-guided AIIS injection and received subspinal decompression in arthroscopy. Follow-up of these 73 patients is still ongoing for assessing the long-term effect of our diagnosis method.

It is worth noting that only 53.49% of patients with Type IIA AIISs and 64.71% of patients with Type IIB AIISs reported a positive response. Additionally, one of three patients reported a negative response after the ultrasound-guided AIIS injection among those with Type III AIISs. Our diagnostic method confirms the conclusion drawn by Balazs et al. [22] and Wong et al. [12]; that is, subspinous impingement is not completely due to an abnormal morphology of the AIIS. In addition, Balazs et al. thought to diagnose SSI according to the AIIS morphology, but it had high sensitivity and extremely low specificity (23%) [22]. If our diagnostic method is used as a standard, the specificity of classification proposed by Hetsroni et al. was only 27.03%. A similar conclusion was also reported by Karns et al., who found that AIIS Types I and II were hard to subdivide and showed poor correlation with the perioperative findings. Therefore, Type II AIIS possibly represents normal AIIS morphology variants rather than pathological abnormalities [23].

Another point demonstrated by our study is that it is inaccurate to assess symptomatic subspine impingement according to whether the patient has superior capsular oedema on MRI. Previously, Larson noted that soft tissue hypertrophy over the medial femoral neck and synovial oedema can be observed frequently in the setting of subspine impingement [24]. Samim et al. first determined the MRI findings associated with SSI and found that the occurrence of superior capsular oedema (75% vs. 7.1%), impingement of the distal femoral neck (90% vs. 16%), superior chondral lesions (80% vs. 45%), and distal cam (80% vs. 19%) was much higher in the SSI group than in the non-SSI group [13]. However, according to our diagnostic method, this difference is not well reflected. Although there was a significant difference in percentage of patients with superior capsular oedema between the two groups (*P =* 0.036), superior capsular oedema was only reported in 38.89% of patients who reported a positive response and was not observed in any of the patients with a Type III AIIS. After evaluating the result reported by Samim et al., Guermazi proposed that possible overlapping impingement syndromes had not be considered, which meant the source of the superior capsular oedema was uncertain [25]. The results of our study suggested that the signs of impingement on the distal femoral neck, such as superior capsular oedema, have a certain level of suggestiveness but cannot be used for diagnosis due to their low sensitivity and specificity.

SSI is usually identified as distal femoral neck contact of the AIIS in full hip flexion [13, 26]. In recent years, Karns et al. believed that the possibility of direct impingement may be very small, and the occurrence of SSI was likely due to the reduction or disappearance of the space under the anterior inferior iliac spine [23], challenging the diagnostic criteria mentioned above. In general, our study indicates that the occurrence of subspine impingement can be heterogeneous. If the subspinal decompression is only determined by anatomical morphology and soft tissue changes, missed diagnosis or unnecessary damage to the physiological structure of patients are possible.

### Limitations

Our study has several limitations. First, a universally acknowledged and unchallenged consensus or gold standard in the diagnosis of SSI is currently lacking. In view of this, we are unable to design a control group to provide more statistical support for our diagnostic method of ultrasound-guided AIIS blocking to diagnose SSI and determine the sensitivity and specificity. We expect to obtain long-term patient follow-up results to provide more evidence supporting our diagnostic method. Second, our study only included a small number of patients, especially patients with Type III AIISs. We recommend further research to focus on this rare group of patients.

## Conclusion

Radiography, including 3-D CT and MRI, as well as the intraoperative findings of the labrum, cannot be considered an accurate and reliable diagnosis and treatment bases for symptomatic SSI in FAI patients. It is suggested that ultrasound-guided AIIS injections be combined with radiography to better diagnose SSI.

## Data Availability

The datasets generated and/or analysed during the current study are not publicly available due to patient privacy concerns but are available from the corresponding author on reasonable request.
